# Influence of chemically p-type doped active organic semiconductor on the film thickness versus performance trend in cyanine/C_60_ bilayer solar cells

**DOI:** 10.1088/1468-6996/16/3/035003

**Published:** 2015-05-14

**Authors:** Sandra Jenatsch, Thomas Geiger, Jakob Heier, Christoph Kirsch, Frank Nüesch, Adriana Paracchino, Daniel Rentsch, Beat Ruhstaller, Anna C Véron, Roland Hany

**Affiliations:** 1 Empa, Swiss Federal Institute for Materials Science and Technology, Laboratory for Functional Polymers, CH-8600 Dübendorf, Switzerland; 2 Institut des Matériaux, Ecole Polytechnique Fédérale de Lausanne, EPFL, Station 12, CH-1015 Lausanne, Switzerland; 3 Zürich University of Applied Sciences, Institute of Computational Physics, Technikumstrasse 9, CH-8401 Winterthur, Switzerland

**Keywords:** cyanine dye, doping, organic solar cell, bilayer

## Abstract

Simple bilayer organic solar cells rely on very thin coated films that allow for effective light absorption and charge carrier transport away from the heterojunction at the same time. However, thin films are difficult to coat on rough substrates or over large areas, resulting in adverse shorting and low device fabrication yield. Chemical p-type doping of organic semiconductors can reduce Ohmic losses in thicker transport layers through increased conductivity. By using a Co(III) complex as chemical dopant, we studied doped cyanine dye/C_60_ bilayer solar cell performance for increasing dye film thickness. For films thicker than 50 nm, doping increased the power conversion efficiency by more than 30%. At the same time, the yield of working cells increased to 80%. We addressed the fate of the doped cyanine dye, and found no influence of doping on solar cell long term stability.

## 1. Introduction

Planar organic electron donor–acceptor (D–A) bilayers have been widely used to study fundamental optoelectronic processes in organic solar cells (OSC). In particular, the heterojunction interface which predominantly determines the charge carrier separation efficiency and recombination losses can be studied and modified much more easily in bilayer devices than in the usually higher performing bulk heterojunction OSC [1]. In combination with the control of crystallinity and morphology of the separate layers the power conversion efficiency could be improved in many systems [2–5]. Unlike in polymer/fullerene blends, an issue arises when using small molecules in bulk heterojunction devices. In D–A small molecule blend films, the marked tendency to crystallization may lead to strong phase segregation resulting in reduced D–A interfacial area, thus decreasing charge generation [6, 7].

A drawback of the bilayer structure is that the effective layer thickness is restricted to the exciton diffusion length (*L*_ex_), since any light that is absorbed at a distance longer than *L*_ex_ from the heterojunction does not contribute to charge generation [8]. Furthermore, most disordered organic semiconductors have hole mobility values in the range of 10^−5^–10^−6^ cm^2^ V^−1^ s^−1^ which limits charge extraction in both bilayer and bulk heterojunction devices. However, only in the former, an s-shaped current–voltage curve may occur for thick donor layers because of the low hole mobility [9, 10]. The constraints of small *L*_ex_ and a low hole mobility value in organic semiconductors often resulted in experimental layer thicknesses of well below ∼50 nm for optimized bilayer OSC. However, for such thin films the probability of pinholes and shorts is higher and consequently the device processing yield is low. This becomes particularly critical when thin films have to be coated on rough substrates or when the device area is increased [11].

It has been shown that the intrinsically low conductivity of small molecules and polymers in OSC applications can be improved by orders of magnitude by chemical doping [12, 13]. Related to the increased conductivity the Fermi level within the organic layer can be adjusted precisely [14]. P-type doping can be realised with various materials ranging from small molecules (such as tetrafluoro-tetracyanoquinodimethane, F_4_-TCNQ [15]), to oxides (e.g. WO_3_ [16]), and salts (such as NOBF_4_ [17]). However, only a few of these materials are compatible with solution processing, are inherently stable and do not initiate undesirable side reactions. Recently, Burschka *et al* presented the use of a Co(III) complex (FK102) as an efficient p-type dopant for spiro-MeOTAD in dye-sensitized solar cells [18]. This complex has a good solubility in highly polar solvents, is inherently stable and has a redox potential of 5.54 eV which allows for efficient doping of several donor materials. The complex exhibits strong absorption in the UV region (ligand centred *π*–*π*∗ transitions) and only a very weak d–d transition in the region between 400–600 nm, having a molar extinction coefficient of less than 30 and 100 l mol^−1^ cm^−1^ in the case of Co(II) and Co(III) species, respectively. Therefore, filtering of incoming light by the dopant is avoided. We mention that Mendéz *et al* recently presented a second condition for efficient doping. Beside the requirement of close-lying electron affinity of the dopant and ionization energy of the organic material, also the intermolecular resonance integral between the dopant and the donor should be as small as possible [19]. Chemical doping has also been used for buffer layers in organic light emitting diodes and OSCs. The increased conductivity was exploited to tune the optical light outcoupling and incoupling, respectively, by the possibility of using thicker organic hole and electron transport layers [15]. Furthermore, the Fermi level adjustment enables the formation of Ohmic contacts to the metal electrodes [20]. Even contacts not containing indium tin oxide (ITO) were presented using the technique of doping [21].

P-type doping with oxygen and NOBF_4_ has been performed on cyanine/C_60_ bilayer solar cells [17, 22]. In both cases, the figures of merit of solar cells were improved by adding the dopant to the donor material. However, it was reported that neither dopant is easily applicable. Oxygen doping is difficult to control and it partly destroyed the film. On the other hand, NO^+^ is very reactive and prone to side reactions. From ^19^F and ^31^P nuclear magnetic resonance (NMR) spectra, we detected the immediate formation of several decomposition products when dissolving NOPF_6_ in acetonitrile under inert atmosphere [23]. Gradually, the solution became unstable and the dopant reacted with the solvent.

Here we report on the use of the Co(III) complex FK102 as p-type dopant for a heptamethine cyanine dye and on the performance of corresponding doped cyanine/C_60_ solar cells. This dopant is easy to handle and inherently stable when dissolved in acetonitrile over a period of more than twelve weeks, as measured by NMR spectroscopy. We show that the conductivity of the cyanine can be increased by more than two orders of magnitude upon dopant addition. The influence on solar cell performance was studied for different doped cyanine layer thicknesses. Even if the limitation of *L*_ex_ is not superable by doping, the developing s-shape for thicker cyanine layers due to the low hole mobility could be overcome by doping. Moreover, we investigated the stability of the doped species in film and solution and discuss their influence on device stability.

## 2. Experimental details

For solar cell fabrication, glass/ITO substrates (Geomatec, sheet resistance 20 Ohms square^−1^) were consecutively cleaned in acetone, isopropanol, detergent and deionised water. The substrates were transferred to a nitrogen-filled glove box and a 10 nm MoO_3_ hole extraction layer (Sigma Aldrich, 99.99%) was vapour-deposited. The donor material Cy7-P was synthesized as described previously [24]. The dopant tris(2-(1H-pyrazol-1-yl)pyridine)cobalt(III) tri(hexafluorophosphate) (FK102, Dyenamo) and Cy7-P were dissolved separately in acetonitrile (Sigma-Aldrich, anhydrous >99.8%) and mixed according to the stated molar ratio prior to spin coating. The thickness of this layer was varied using different solution concentrations (1.0–15 mg ml^−1^ acetonitrile) while the spin coating speed was fixed to 6000 rpm. The thickness of Cy7-P films was determined by comparing with absorption spectra of reference samples on glass. Film thicknesses of reference samples were determined by profilometry (Ambios XP1). The error in the thickness due to batch-to-batch variation was less than 5 nm. To complete the bilayer a 40 nm thick C_60_ (SES Research, 99.9%) film was thermally evaporated. The top electrode consisted of a 2 nm buffer layer of tris(8-hydroxyquinolinato)aluminium (Alq_3_, Sigma Aldrich, 99.995%) and Ag which was evaporated through a shadow mask to define cells with an active area of 3.1 and 7.1 mm^2^. Average performance values for undoped cells with active layers <30 nm were obtained for 2–4 cells, whereas 7–42 cells were measured for thicker films.

Solar cells were characterized under inert atmosphere on a calibrated solar simulator (Spectra Nova) using a Xe lamp with 100 mW cm^−2^ simulated AM1.5G solar irradiation. The light intensity was adjusted using a calibrated silicon reference cell from Rera Solutions and no corrections for the spectral mismatch were applied. Incident photon-to-current conversion efficiency (IPCE) was measured using a monochromator and a 300 W Xe light source equipped with an AM1.5G filter. The measurement was performed without additional bias light. Absorption measurements were performed on a Varian Cary 50 UV–vis spectrophotometer. Fluorescence spectra were measured on a Horiba Jobin Yvon Fluorolog spectrometer. For conductivity measurements Au contacts were evaporated on glass/Cy7-P samples with defined distances of 0.2, 0.5, 1 and 2 mm. By measuring the conductivity for different channel lengths the effect of the contact resistance could be separated. For these measurements, the Cy7-P film thickness was 72 nm. Conductivity samples were prepared and stored under nitrogen atmosphere but the measurements were performed in ambient atmosphere. Electro-optical simulations were carried out with the program SETFOS, version 4.1 (Fluxim). NMR spectra were measured on a 400 MHz Avance III spectrometer.

## 3. Results and discussion

### 3.1. Intrinsic cell performance

**Figure 1.**
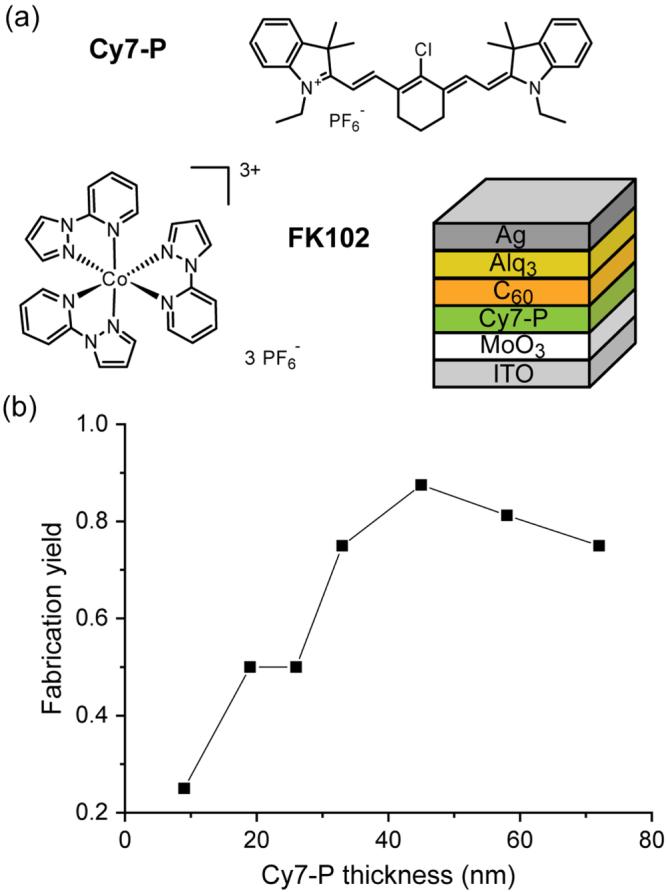
(a) Chemical structure of the cyanine donor Cy7-P and the dopant FK102, as well as a sketch of the regular device structure. (b) Yield of working cells as a function of Cy7-P thickness.

**Figure 2.**
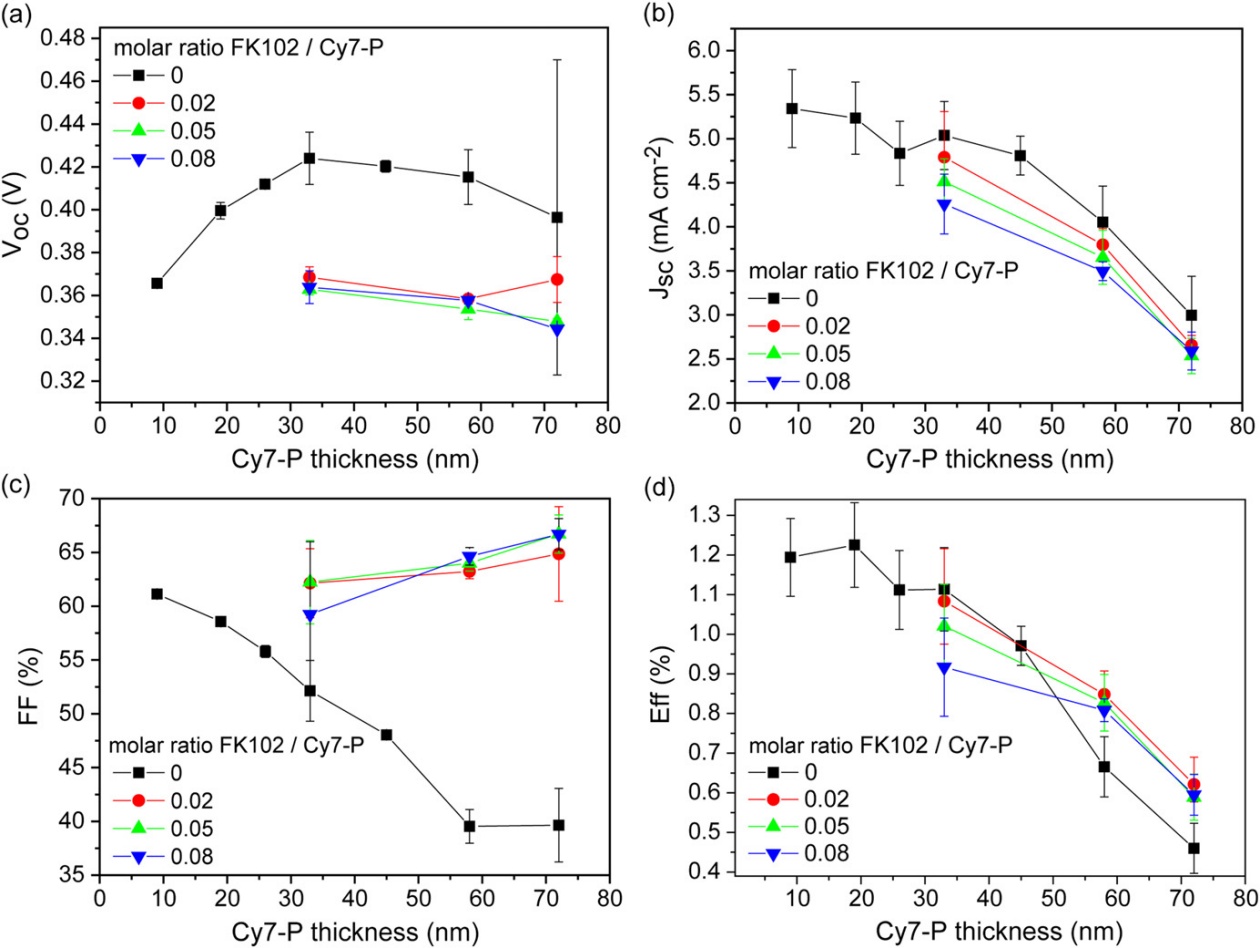
Measured (a) open circuit voltage (*V*_oc_), (b) short circuit current density (*J*_sc_), (c) fill factor (FF) and (d) power conversion efficiency (Eff) of undoped (black squares) and FK102 doped Cy7-P cells as a function of Cy7-P layer thickness and doping concentration.

Bilayer solar cells with the structure presented in figure 1(a) were fabricated with different Cy7-P thicknesses ranging from 9 to 72 nm. As shown in figure 1(b) the yield of working devices was only about 20% for the thinnest layer and increased with film thickness. Therefore, the use of cells with Cy7-P layers thicker than 30 nm is desirable. In figure 2 the figures of merit for undoped cells (black squares) are presented as a function of Cy7-P thickness. For thicknesses above 30 nm a slight decrease of the open circuit voltage (*V*_oc_) from 0.42 V (33 nm) to 0.40 V (72 nm) was observed, whereas a *V*_oc_ loss of 20% occurred when the Cy7-P layer thickness was reduced to 10 nm. The second effect can probably be attributed to the non-continuous film formation of such thin layers on MoO_3_. Thus, evaporated C_60_ is in direct contact with MoO_3_ which leads to increased recombination and consequently to a lowered *V*_oc_ [25, 26]. The *V*_oc_ decrease for thick layers is attributed to increased recombination because of a reduced effective electric field within the Cy7-P layer. This trend could be reproduced in simulations by only changing the active layer thickness (see supplementary data S1).

Within the accuracy of the measurement the short circuit current density (*J*_sc_) stayed constant up to a thickness of 40 nm, above this value the current decreased dramatically with increasing Cy7-P layer thickness. This behaviour is attributed to two different effects. Firstly, in layers which are thicker than the exciton diffusion length (*L*_ex_) a fraction of the impinging light is absorbed too far away from the interface and does not contribute to the charge generation. This parasitic absorption of Cy7-P cannot be avoided in regular devices, and only the light that is absorbed within *L*_ex_ generates charges. Secondly, charge carriers that are created at the heterojunction interface have to travel through the whole Cy7-P layer to the hole collecting contact. Since the built-in voltage and the hole mobility do not change by varying the thickness, the effective electric field that the charges feel is lower for thicker layers. A space charge region is formed for thicker layers at the interface and thus, a higher amount of free charges will recombine with electrons at the heterojunction interface. An indication that charge carrier extraction is less efficient for increasing Cy7-P layer thickness can be seen in the steady decrease of the FF. The trend of FF and *J*_sc_ for thick layers is qualitatively well reproduced by simulations if the only parameter that is varied is the thickness of Cy7-P (see supplementary data S1). Thin films are more challenging to model, as e.g. effects of a non-continuous surface coverage are not taken into account. The efficiency peaks for ∼20 nm thick films and decreases by ∼60% when the layer thickness is increased to 72 nm. Best performances are in good agreement with data presented elsewhere, using different coating solvents for Cy7-P [24].

### 3.2. Doping

**Figure 3.**
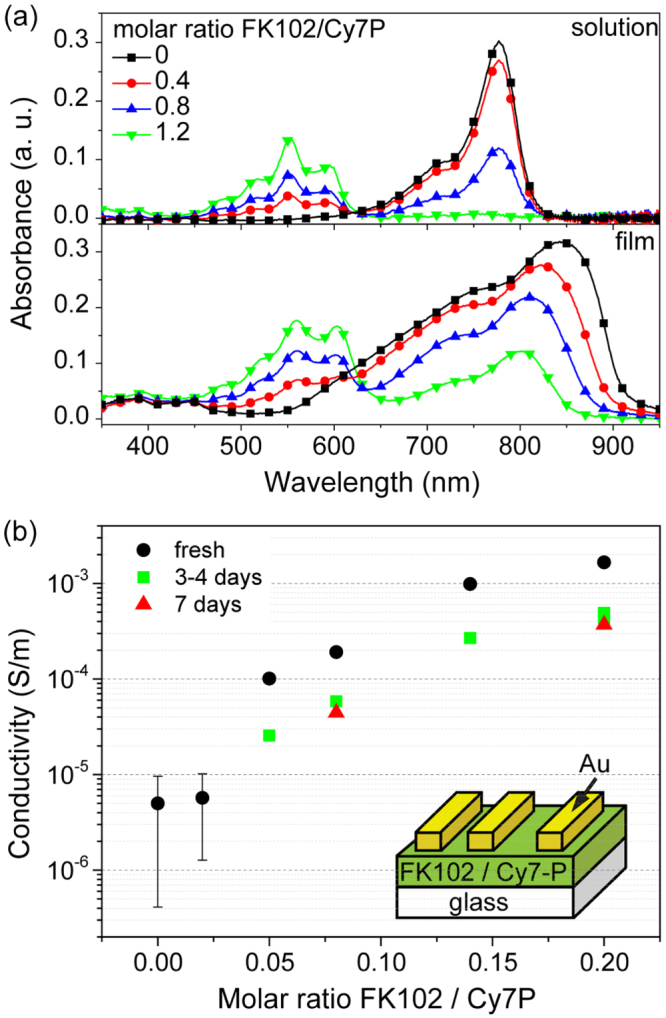
(a) UV–vis spectra of doped solutions and films with molar doping ratios of 0 (black squares), 0.4 (red circles), 0.8 (blue up triangles), 1.2 (green down triangles). Solution spectra were measured by compressing a thin liquid film (1 mg Cy7-P ml^−1^ solvent) between two glass plates. (b) Conductivity as a function of doping level. Samples were either measured directly after coating (black circles) or were stored for 3–4 days (green squares) and 7 days (red triangles), respectively, before Au electrodes were evaporated and the conductivity was measured. Inset: structure of the device used for conductivity measurements.

The driving force for p-type doping of Cy7-P by FK102 is around 0.24 eV, assuming a HOMO level of 5.3 eV for Cy7-P [24] and a redox potential of 5.54 eV for the dopant [18]. The effect of doping was investigated in three different experiments. Firstly, doped solutions and films were analysed by absorption measurements. In order to clearly identify spectral variations, high molar doping ratios (10 times higher than in solar cells) had to be chosen. Figure 3(a) shows absorption spectra for solutions and coated films of untreated and doped samples. The pure dye in solution showed a peak at 777 nm. The absorption spectrum of a spin-coated Cy7-P film was considerably broadened and extended to longer wavelengths (peak absorption at 842 nm). This can be explained by strong intermolecular interactions and increased molecular order in the solid state. By adding more and more dopant the Cy7-P peak decreased and a new peak appeared with a maximum absorption at 553 nm in solution and at 560 nm in the film. This peak is attributed to the oxidized Cy7^2^^+·^-P radical cation [27]. In both spectra an isosbestic point can be identified around 620 nm. This is an indication that upon adding the dopant one species is transformed into another one. In solution it was possible to achieve a full transformation to the doped species, from which the maximum extinction coefficient of the oxidized species could be estimated to be ∼120 000 l mol^−1^ cm^−1^, similar to values reported in literature [27]. In the film, the dopant seems to disrupt the interactions between cyanine molecules, resulting in a ∼40 nm blue-shift of the Cy7-P absorption for the highest doping concentration (figure 3(a)). Note that the transformation is nonlinear with increasing doping concentration. This can be explained by side reactions with unspecified impurities in the starting materials or the solvent.

Conductivity measurements were performed on Cy7-P films with different amounts of dopant (figure 3(b)). The conductivity of the undoped and the 2% doped device was close to the detection limit of the setup, therefore, these values are only an upper limit. Increasing the doping ratio led to a pronounced increase in conductivity, larger than two orders of magnitude upon addition of 20% dopant. This super-linear relationship with doping has been observed for various phthalocyanines as well such as F_4_-TCNQ:ZnPc [15, 28] and F_4_-TCNQ:VOPc [29]. The steep increase for low doping concentrations has been explained by trap filling, which implies a rise of the effective mobility and thus the super-linear increase of the conductivity upon doping [15, 30]. The intrinsic conductivity for Cy7-P is in good agreement with values reported for vapour-deposited phthalocyanines [28, 29]. Hole mobilities obtained by photo-charge extraction by linearly increasing voltage measurements have been reported for similar materials [10, 26]. Assuming a value of 10^−5^ cm^2^ V^−1^ s^−1^, the intrinsic charge carrier density in undoped Cy7-P is around 3 × 10^16^ cm^−3^. This value is in good agreement with typical free carrier densities in molecular semiconductors [31].

**Figure 4.**
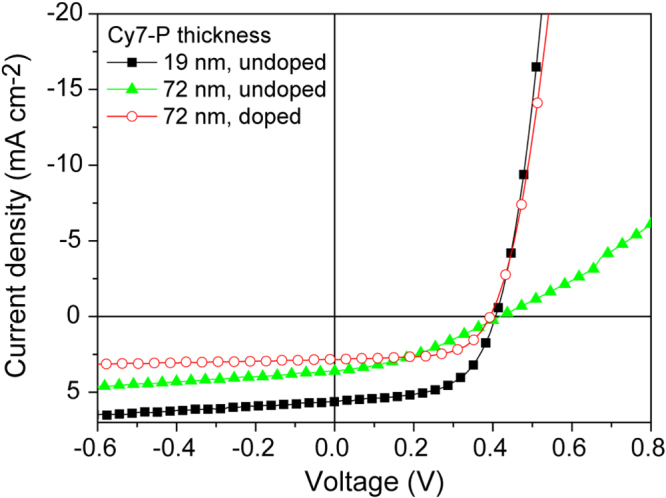
Current–voltage characteristics of the best performing device with a Cy7-P layer thickness of 19 nm (black squares), of an undoped 72 nm thick device (green triangles) and a doped (FK102/Cy7-P = 0.02) 72 nm thick cell (red circles).

Doping concentrations for OSC devices were chosen between 2% and 8% and three different Cy7-P thicknesses were used for comparison (figure 2). Doped cells exhibit strongly increased fill factors. Figure 4 shows the current–voltage characteristics for the best performing cell with a Cy7-P thickness of 19 nm in comparison to the 72 nm thick device with and without 2% doping. Best FFs in doped devices were above 65% for the thickest Cy7-P films (figure 2(c)). Although the conductivity increased by a factor of ten when comparing 2% with 8% doped films (figure 3(b)), there is no clear trend that higher doping concentrations correlate with higher FFs. *V*_oc_ was reduced by doping, but again no systematic variations of *V*_oc_ were found for different doping concentrations (figure 2(a)). The lowering of *V*_oc_ due to doping is attributed to increased charge recombination. Transient photocurrent experiments support this hypothesis (see supplementary data S2).

**Figure 5.**
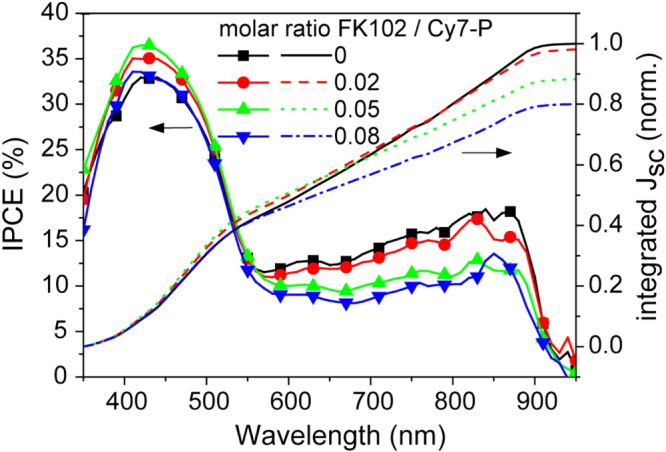
Left axis: IPCE of cells ITO/MoO_3_/Cy7-P (33 nm)/C_60_/Alq_3_/Ag with molar doping ratios FK102/Cy7-P of 0–8%. Right axis: integrated short circuit current from the IPCE data.

For *J*_sc_ a steady decrease as a function of doping concentration was observed for Cy7-P layer thicknesses of 33 and 58 nm, for the thickest film (72 nm) there was almost no difference between different doping concentrations (figure 2(b)). Such behaviour has been described by the less efficient extraction of charge carriers due to a field screening effect close to the contacts for unintentional doping in bulk heterojunction cells [32]. In our devices this reason can be excluded because of the pronounced increase in FF and the IPCE results presented in figure 5. If inefficient extraction limits *J*_sc_, IPCE values would be reduced uniformly across the entire spectral range where C_60_ (below ∼550 nm) and Cy7-P (above ∼550 nm) absorb. An extraction problem would decrease conversion rates independent of the layer where the exciton is generated. However, a clear reduction upon doping is only observed in the wavelength region where Cy7-P absorbs. On the other hand, a reduced charge generation yield in the Cy7-P layer upon addition of FK102 explains the trend in *J*_sc_ and IPCE at the same time. Quenching of excitons at doping sites is a well-known phenomenon, for example in the field of light-emitting electrochemical cells [33, 34]. Thus, with increasing doping, less and less excitons are created in the Cy7-P layer that can reach the heterojunction interface to create free charges and contribute to *J*_sc_. Note that the Cy7^2^^+·^-P is blue-shifted with respect to the non-oxidized Cy7-P and the spectral overlap is very small, thus quenching due to Dexter- or Förster transfer is energetically not favourable and quenching due to direct charge transfer is more likely [34]. In principle, exciton quenching in doped films is detectable via a decrease of the photoluminescence. However, the fluorescence of Cy7-P is very weak and was below the detection limit of our spectrometer even for undoped dye films.

Short circuit current values in figure 2(b) showed a similar trend for undoped and doped cells, but the reasons for the decrease in *J*_sc_ with increasing dye film thickness are distinctly different. In both cases a fraction of the current decrease comes from the constraint that only light absorbed within *L*_ex_ generates free charge carriers. For undoped cells and with increasing film thickness, the current is further decreased due to the low conductivity that hinders charge extraction. For doped cells, holes can be extracted through thicker Cy7-P films, but in this case exciton quenching seems to reduce the charge generation yield.

Efficiency values of doped cells are also shown in figure 2(d). For 33 nm thick dye films, doping adversely affected the overall performance compared to undoped devices, due to the decrease of *V*_oc_ and *J*_sc_. For this film thickness, the decreasing trend of *J*_sc_ with doping is also reflected in the device performance trend. However, for thicker films the efficiencies for doped cells are higher by 30–40%, which can be attributed mainly to the strong increase of the FF. This clearly demonstrates the beneficial effect of doping of thick active layers. Considering the mean errors, there is no apparent trend that smaller doping concentrations increase the performance. We ascertained this for 58 nm thick dye films and measured unchanging performances when decreasing the doping level to 1%.

### 3.3. Stability

**Figure 6.**
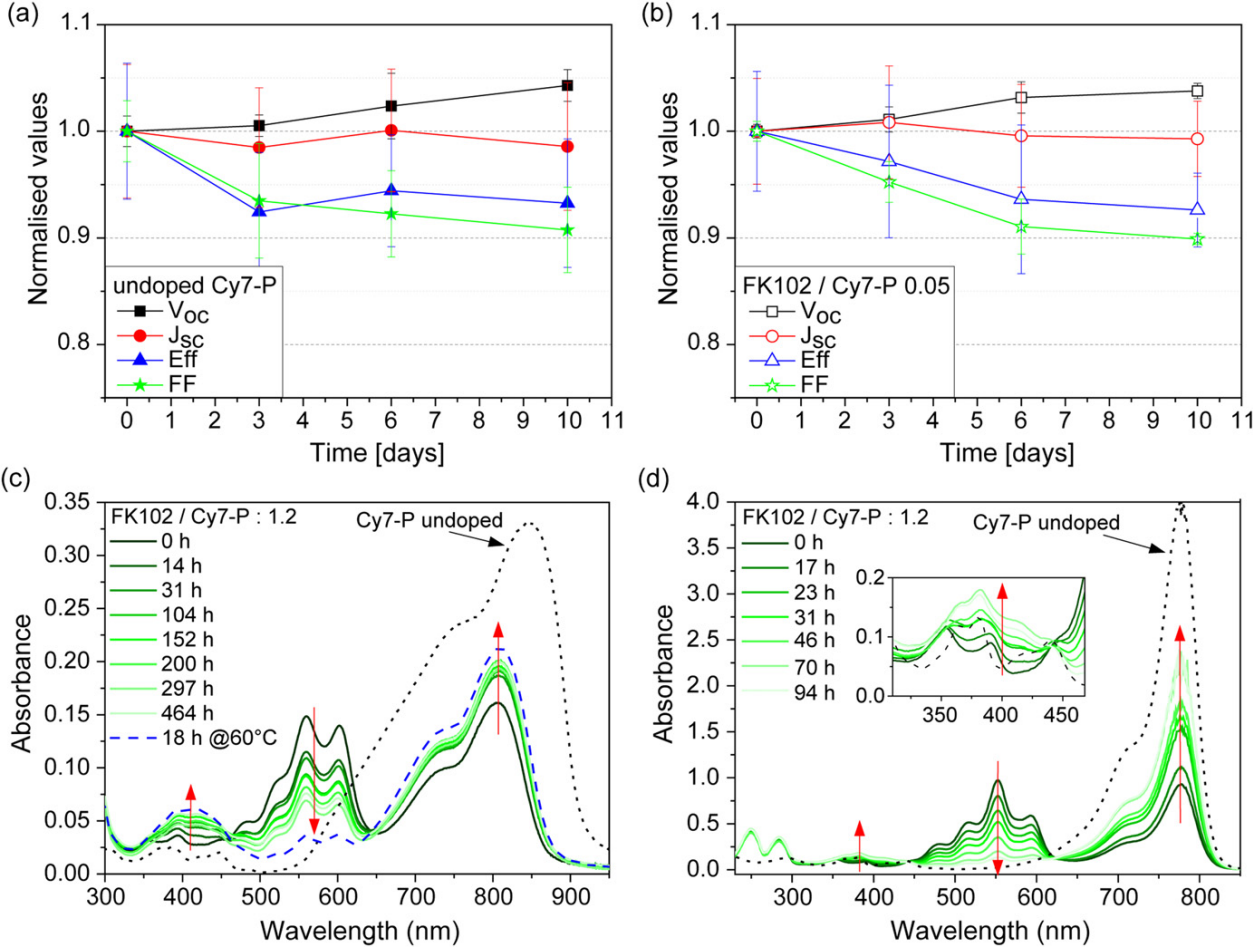
Stability of (a) undoped and (b) doped cells with a Cy7-P thickness of 33 nm stored at room temperature in the dark under nitrogen. (c) Absorption spectra of highly doped films (FK102/Cy7-P = 1.2 mol mol^−1^) stored in the dark under nitrogen (solid green lines). The time evolution is indicated by red arrows. The black dotted line shows the absorption of the undoped film with the same thickness which did not change over time. A film that was stored for 18 h at 60 °C in the dark under nitrogen is depicted as blue dashed line. (d) Absorption spectra of 1 mg ml^−1^ Cy7-P solutions with the same doping concentration as in (c). For every measurement a fraction of the stock solution stored in the dark under nitrogen was removed, diluted to 0.01 mg ml^−1^ under nitrogen and measured immediately in a 1 cm thick quartz cuvette. The time evolution is indicated with red arrows. The absorption spectrum of the undoped solution with the same concentration is shown as black dotted line.

Data in figure 3(b) show that the conductivity of doped films decreased with time, indicating the occurrence of de-doping. Independent of the doping concentration, the conductivity dropped by ∼70% within the first three days. The decay levelled off for longer storage times and the conductivity of aged, doped films remained always higher than the one of undoped dye films. To study the influence of de-doping on OSC performance, undoped (figure 6(a)) and 5% doped (figure 6(b)) devices were stored over a period of 10 days in nitrogen atmosphere and periodically measured. In both cases, an efficiency decrease of ∼6% was found, related to a decrease in FF by about 10%. FF and efficiency remained constant for samples that were stored at −25 °C (see supplementary data S3).

These results show that the drop in conductivity of doped films over time is probably not related to the small performance decay. Still, the fate of the oxidised radical dication Cy7^2^^+·^-P is of importance to address the long-term stability of cyanine dye-based solar cells in general. The radical dication is also formed after light absorption and electron transfer to C_60_. Although the steady state concentration during illumination (∼5 × 10^16^ cm^−3^) is much smaller than the concentration produced by chemical doping (∼7 × 10^19^ cm^−3^, *ρ*(Cy7-P) = 1.519 g cm^−3^ [24]), locally high Cy7^2+^^·^-P concentrations can be expected, for example, in light-emitting electrochemical cells [35] or when holes are retained at energetic barriers.

The stability of cyanine radical dications has been studied in solution [27, 36–38]. The main absorption band of the radical dication is hypsochromically shifted, and the molar extinction coefficient is about one-half of that of the parent dye molecule. Some cyanine radical dications also exhibit weak absorptions in the near-infrared wavelength region [39]. Radical dications are susceptible to radical–radical dimerization at the polymethine chain. The rate of dimerization and stability of the dimer depend on the nature of the heterocyclic unit and the type of alkyl substituent at the methine carbons. In absence of alkyl substituents the dimerization can be irreversible, while their presence stabilizes the radical dication and/or sterically hinders the bimolecular coupling reaction, resulting in reversible dimerization. The tetra-cationic dimer absorbs in the UV spectral region. The methine protons at the coupling positions are acidic, and deprotonation can form a bis-dye that can be further oxidised via a reversible two-electron mechanism to yield a cross-conjugated tetra-cationic species.

We studied the stability of Cy7^2^^+·^-P by recording absorption spectra of doped films (figure 6(c)) and solutions (figure 6(d)) over a period of 5–20 days. Doping concentrations used for OSC were too small to provide spectral information, and highly doped (FK102/Cy7-P = 1.2 mol mol^−1^) solutions were used for these measurements. The absorption spectra of undoped Cy7-P solutions and films did not change over time. In the absorption spectra of doped Cy7-P two isosbestic points are observed around 460 and 650 nm in the film and 440 and 620 nm in solution, respectively. Cy7^2^^+·^-P absorbs between 500 and 600 nm, and the absorption at around 400 nm can be assigned to the dimerization product between two radical dications [27, 36–38].

Both experiments showed a decrease of the Cy7^2^^+·^-P absorption over time with a partial regeneration of the parent dye and an increase of the dimer. However, timescales and redistribution fractions are distinct for film and solution. In the film the Cy7^2^^+·^-P absorption is reduced to 50% of its initial height within 20 days while the parent dye absorption increases by 20% and the dimer absorption doubles its height. This evolution could be accelerated by increasing the temperature, as indicated by the blue dashed line in figure 6(c). In the films that were stored at −25 °C there was no change at all in the absorption spectrum over a period of one week.

In solution with a Cy7-P concentration of 1 mg ml^−1^ the radical dication vanished within 5 days. Again, Cy7^2^^+·^-P dimerized and the parent dye was regenerated to a considerable amount. To investigate the reaction order of the Cy7^2^^+·^-P decay the evolution was measured for different solution concentrations (0.1, 1 and 5 mg ml^−1^). Since there are at least two products involved the analysis is not straightforward. However, it was found that the half-life of Cy7^2^^+·^-P depends on its concentration which excludes a monomolecular reaction. The described transformations with similar timescales were also observed if Cy7-P was doped by NOPF_6_ (see supplementary data S4). This excludes the transformation to be dependent on the dopant’s chemical structure, redox potential and electronic coupling to Cy7-P.

Note the differences between UV–vis spectra shown in figures 3(a) and 6(d). A full transformation to the doped species was observed when a highly concentrated solution was compressed as a thin liquid film between two glass plates (figure 3(a)) while pristine Cy7-P is still present directly after preparing the doped solution in figure 6(d). The partial doping is due to side reactions with impurities and residual water in the solvent acetonitrile that was added to dilute the highly concentrated solution (1 mg ml^−1^) for more reproducible UV–vis measurements in the quartz cuvette (0.01 mg ml^−1^). We also observed that doped solutions are very sensitive to ambient atmosphere and the Cy7^2^^+·^-P species disappeared over a period of one hour when storing a diluted solution outside the glovebox.

The timescale of many hours over which the radical dication decays under inert conditions indicates that Cy7^2^^+·^-P is remarkably stable. This can tentatively be ascribed to the cyclohexenyl ring that stabilizes the radical [27, 36–38]. In addition, the dimethyl groups on the indole moiety sterically shield the methine carbons adjacent to the heterocyclic nuclei and hinder the dimerization. The Cy7^2^^+·^-P stability is further demonstrated by the reversible cyclic voltammograms [24]. Unsubstituted cyanine dyes often show irreversible oxidation and reduction, even at high scan rates. In an interesting approach, the stability of an unsubstituted heptamethine cyanine dye could be greatly increased by rotaxane encapsulation [39].

A mechanism of radical dication decay that partially regenerates the parent dye has been described [38]. It involves the loss of a proton of cyanine^2+ ·^ to form the corresponding cation radical, cyanine^+ ·^ . In a second step, cyanine^+ ·^ undergoes a homogeneous electron exchange with another cyanine^2+ ·^ to regenerate one equivalent of the parent dye. From the absorption spectra shown in figures 6(c) and (d), it can be concluded that for high doping concentrations this mechanism is an important decay channel of Cy7^2^^+^^·^-P, and dimerization is occurring especially in solution to a smaller extent. However, from our data it cannot be concluded whether such bimolecular reactions also dominate the radical dication decay in doped OSC devices, or whether alternative (monomolecular) reaction channels become important for smaller doping concentrations.

## 4. Conclusions

As a result of the small exciton diffusion length *L*_ex_ in organic semiconductors, the highest solar cell performance was found for cyanine layer thicknesses of around 20 nm. However, this was accompanied by a low device fabrication yield, even for our small device areas which were well below 1 cm^2^. The limitation imposed by *L*_ex_ cannot be overcome by increasing the film thickness, but our result show that the power conversion efficiency of cells with thicker dye films could be increased considerably by chemical doping. Chemical reasons for the observed conductivity decrease of doped films are difficult to address because of the small doping concentrations used in OSC devices. However, by using higher doping concentrations, we found that the Cy7^2^^+^^·^-P radical dication is remarkably stable. This provides design criteria of how to stabilize cyanine radical dications in general. These species are produced upon chemical p-type doping, but are also formed during solar cell operation or when cyanines are used as sensitizers for silver halide photography, and are present in high concentrations in operating cyanine dye-based light-emitting electrochemical cells.
